# Natural Compounds as Beneficial Antioxidant Agents in Neurodegenerative Disorders: A Focus on Alzheimer’s Disease

**DOI:** 10.3390/antiox8120608

**Published:** 2019-11-30

**Authors:** Antonella Amato, Simona Terzo, Flavia Mulè

**Affiliations:** 1 Department of Biological, Chemical and Pharmaceutical Sciences and Technologies, University of Palermo, 90127 Palermo, Italy; simona.terzo01@unipa.it (S.T.); flavia.mule@unipa.it (F.M.); 2 Department of Neuroscience and Cell Biology, University of Palermo, 90127 Palermo, Italy

**Keywords:** neurodegenerative diseases, Alzheimer’s disease, curcuminoids, silymarin, chlorogenic acid, microalgae, *Aphanizomenon flos aquae*

## Abstract

The positive role of nutrition in chronic neurodegenerative diseases (NDs) suggests that dietary interventions represent helpful tools for preventing NDs. In particular, diets enriched with natural compounds have become an increasingly attractive, non-invasive, and inexpensive option to support a healthy brain and to potentially treat NDs. Bioactive compounds found in vegetables or microalgae possess special properties able to counteract oxidative stress, which is involved as a triggering factor in neurodegeneration. Here, we briefly review the relevant experimental data on curcuminoids, silymarin, chlorogenic acid, and compounds derived from the microalga *Aphanizomenon flos aquae* (AFA) which have been demonstrated to possess encouraging beneficial effects on neurodegeneration, in particular on Alzheimer’s disease models.

## 1. Introduction

Chronic neurodegenerative diseases (NDs), such as dementia and its most frequent etiological types—Alzheimer’s disease (AD) and Parkinson’s Disease (PD), are a growing cause of disability and death, characterized by progressive loss or dysfunction of neurons in specific areas of the brain. Patients with these disorders display variable clinical features including cognitive decline, speech difficulties, and motor impairment [1]. In particular, AD is primarily a dementia-related disorder characterized by a progressive decline in cognitive function and memory [2], while PD is primarily a movement disorder illness with four major symptoms: tremor, slowness of movement, muscle rigidity, and postural instability.

Neurodegenerative diseases represent a great public health concern and socioeconomic problem because of the high mortality rates and healthcare costs for treatment and care assistance. Furthermore, the current therapies for the treatment of AD and PD are only able to reduce the symptoms and cannot arrest the development of neurodegeneration [3]. Therefore, more research needs to be conducted to better understand the biochemical cascades of events resulting in neurodegeneration and to discovery potential new neuroprotective drugs able to improve neuronal cell loss and restore brain normal functioning.

Although AD and PD pathophysiology presents marked differences, some biochemical reactions leading to neurodegeneration are shared by both diseases. The major pathological feature of AD includes formation of amyloid plaques in extracellular spaces, caused by the deposition of the amyloid-β (Aβ) peptide, derived by proteolytic cleavage of the amyloid protein precursor (APP), and intracellular neurofibrillary tangles (NFTs) [4,5]. Intracellular neurofibrillary tangles are composed of hyperphosphorylated Tau proteins in neurons located particularly in the hippocampus and cerebral cortex regions of the brain. The reduced expression of the brain-derived neurotrophic factor (BDNF), important for neuronal growth and memory functions, also plays a crucial role in AD pathogenesis via formation of senile plaque of Aβ and NFTs. At the molecular level, Aβ induces oxidative stress, inflammation, and mitochondrial dysfunction associated with apoptosis [6,7].

Parkinson’s disease is characterized by the progressive loss of dopaminergic neurons in the substantia nigra (SN) and formation of Lewy bodies, abnormal intraneuronal aggregates of proteins that include α-synuclein, ubiquitin, and neurofilaments [8]. The pathogenic mechanisms of PD include oxidative stress, mitochondrial and protein dysfunction, inflammation, and apoptosis [9]. In particular, oxidative stress generates ROS which activates glial cells, and activated glial cells generate inflammation that results in mitochondrial dysfunction, and, consequently, dysfunctional mitochondria activate cell death machinery [10].

Hence, oxidative stress and mitochondrial damage are common factors involved in the etiopathology of these neurodegenerative diseases [11,12,13].

### 1.1. Oxidative Stress and Neurodegeneration

Oxidative stress is characterized by an overproduction of ROS—oxygen-containing molecules known to be highly reactive.

Reactive oxygen species are formed in mitochondria during oxidative phosphorylation as a byproduct of normal metabolism. Therefore, modest levels of ROS are normally expected and are considered essential for the regulation of normal cell functions, while excessive ROS production can damage various biological targets, such as DNA, lipids, and proteins, causing serious neurological injury. Because the brain consumes a large amount of molecular oxygen to function properly, the accumulation of damaged biomolecules caused by ROS is high, especially with aging [14]. To limit these cellular damages, brain neurons produce antioxidant defense enzymes, such as superoxide dismutase (SOD), glutathione peroxidase (GPX) and catalase (CAT), which neutralize the damaging effects of ROS accumulation. The proper balance between oxidants and antioxidants can be disrupted with the overproduction of free radicals. Overproduction of ROS in brain cells can lead to free radical attacks against poly-unsaturated fatty acids (PUFAs) at the cell membrane which can, in turn, induce lipid peroxidation [15]. Lipid peroxidation products, such as malondialdehyde (MDA) and hydroxynonenal (4-HNE), are very toxic to neurons and can induce neuronal death [16]. Both AD and PD are usually considered age-related neurodegenerative diseases due to the close relationship between ROS-induced damage and aging.

### 1.2. Diet and Neurodegeneration

Lifestyle factors, such as obesity, physical inactivity, and unhealthy diet, are associated to the increased risk of developing neurodegenerative disorders. In particular, high intakes of carbohydrates, animal-based proteins, and saturated fats can promote increased ROS generation which, in turn, is involved in the onset of obesity-related dysmetabolisms [17] including neurodegeneration [18]. Furthermore, a healthy diet contributes to the physiological development of the central nervous system and participates in the maintenance of neuronal plasticity [19]. On the contrary, a wrong and unbalanced diet can have a severe impact on brain function, thereby contributing to the onset and worsening of neural dysfunctions. A recent work [20] showed that, in the brains of mice fed with a hyperglycemic diet (HGD) or high-fat diet (HFD), changes occurred in the phosphorylation of proteins involved in synaptic plasticity and neuronal functionality. In particular, dephosphorylation of proteins involved in neuronal development (synaptic Ras GTPase-activating protein 1 (SYNGAP1) and protein phosphatase 1 regulatory subunit 9B (PPP1R9B)), vesicle trafficking (clathrin-associated protein (SNAP91) and amphiphysin (AMPH)), and in cytoskeletal functions (cytoplasmic linker-associated protein 2 (CLASP2) and glycogen synthase kinase (3β-GSK3B)) was identified, while increased phosphorylation was detected for microtubule proteins (microtubule-associated protein 2 (MAP2) and microtubule-associated protein Tau (MAPT)). The variation in phosphorylation of vesicle trafficking and cytoskeletal proteins in the brain could be the cause of alterations in neuronal functions, being correlated physiologically to axonal transport and pathologically to neurodegenerative disorders [20]. Then, this study confirms a connection between diet-induced obesity and impairment of neuronal functions and signaling. In agreement, diets rich in fat and sugar (i.e., Western diet) have been linked to the development of neurodegeneration [21], depression, and cognitive decline [22,23]. Indeed, obesity and its comorbidities are considered risk factors for impaired cognitive performance and cognitive decline. Increased body mass index (BMI) and waist circumference are negatively associated with impaired memory performance [24], psychomotor function [25], and selective attention [26]. Obesity is also associated with decreased brain volume [27], in particular grey matter atrophy of the temporal and frontal cortex and hippocampus [28,29] which represent the brain areas mainly affected by morpho-functional damage in the early stage of AD [30].

Some evidence suggests that diet-induced obesity produces high levels of ROS in mice brains [31] which, in turn, are associated with increased levels of AD biomarkers and inflammation [32,33]. Nuzzo and colleagues [18,34] demonstrated that, in HFD mice, obesity was associated with cerebral increase in oxidative stress, mitochondrial dysfunction, APP expression, neuroinflammation, and apoptosis. Another study confirmed that, the brain of HFD-fed obese mice prsent Aβ depositions, dysregulations in neuronal apoptosis and autophagy activity, the latter being a cellular process which allows to sustain cell viability when the exogenous nutrient supply is not available [35]. Several studies [36,37] have reported that in different mouse models, the appearance of insulin resistance causes the suppression of autophagy. A growing body of evidence shows that insulin resistance can play an important role in the pathogenesis of AD [38]. Glucose is the main fuel of the brain, and glucose uptake and utilization are stimulated by insulin. Insulin resistance leads to impairments in glucose metabolism increasing oxidative stress, production of ROS, and mitochondrial dysfunction which drive pro-apoptosis, pro-inflammatory, and pro-Aβ cascades [39]. Accordingly, obesity-related brain-specific insulin resistance, known as type III diabetes [40,41], has been reported in dementia, including AD, suggesting that it may contribute to neurodegeneration [42,43].

## 2. Dietary Natural Compounds and Neuroprotection

Growing evidence shows that certain dietary compounds, with neurogenic properties play a beneficial role in brain aging and neurodegenerative disease [44,45,46,47,48,49]. In particular, compounds such as polyphenols (i.e., flavonoids, curcuminoids, stilbenes, phenolic acids, carotenoids), abundant in various alimentary sources (such as tea, red wine, herbs, seeds, and fruits), represent the phytochemicals underlying the health effects associated to the constant consumption of fruits and vegetables.

Although the molecular mechanisms have yet to be established, polyphenols are able to induce neurogenesis in the brain [49,50,51,52], reduce oxidative stress and neuroinflammation [53,54], and to enhance cell signaling [55]. The potent antioxidant action of polyphenols takes place through scavenging of free radicals and the consequent creation of more stable compounds [56].

A plethora of polyphenols have been investigated in relation to brain health. This review summarizes the neuroprotective activities of curcuminoids, silymarin, and chlorogenic acids, for which we recently explored the beneficial properties in preventing obesity-related neurodegeneration [57]. We delve into the efficacy of these natural compounds to counteract neuronal dysfunctions underlying AD pathogenesis.

### 2.1. Curcuminoids

Curcuminoids are the most important group of chemical components of turmeric, a bright yellow aromatic powder obtained from the rhizome of a plant belonging to the family Zingiberaceae (*Curcuma longa*) and used to give flavor and color in Asian cooking. Curcuminoids include demethoxycurcumin, bisdemethoxycurcumin, and curcumin which are the main polyphenols of *Curcuma* [58].

A large amount of in vitro and in vivo experiments using animal models suggest that curcuminoids can exert neuroprotective effects. The main outcomes are summarized in Table 1.

In vitro studies have shown that curcuminoids are able to restore excitability in hippocampal CA1 neurons injured by exposure to Aβ peptides [59] and that curcumin is able to prevent Aβ-aggregation and oligomer formation [60,61] by directly binding Aβ peptides [62]. Furthermore, curcumin positively influences Aβ cellular uptake [63], preventing plaque deposition and avoiding cellular insults induced by the peptides [64]. It can also downregulate Aβ production by suppressing β amyloid-induced BACE-1 (beta-site APP-cleaving enzyme) upregulation [65]. A recent study has shown that curcumin downregulates the expression of amyloid precursor protein and amyloid-β in swAPP695-HEK293 cells through the increase of miR-15b-5p expression. The microRNA seems to target the 3’-untranslated region of amyloid precursor protein leading to silencing of APP expression [66].

In vivo administration of curcuminoids is able to enhance spatial learning and memory in rats displaying AD-like neurodegeneration [67], while curcumin administration is able to rescue the Aβ-related neurite morphological changes that occur near plaques [68] and to reduce brain Aβ burden as well as inflammation and microglia activation in AD mouse models [69]. Ishrat and colleagues [70] demonstrated that curcumin supplementation decreases cognitive behavior and the biochemical and histopathological alterations in the brain of intracerebroventricular (CV)-streptozotocin (STZ)-infused rats by reducing MDA and H_2_O_2_ levels and increasing the concentrations of glutathione (GSH) and GSH-dependent antioxidant enzymes (glutathione peroxidase and glutathione reductase) in the hippocampus and cerebral cortex [70].

Lim et al. [71] verified that administration of a curcumin diet significantly reduced inflammatory marker expression, such as IL-1β and glial fibrillary acidic protein (GFAP), and oxidized the protein concentration and the overall insoluble Aβ, soluble Aβ, and plaque burden in the brains of Alzheimer transgenic APP mouse model [71]. Moreover, curcumin intake was shown to reduce amyloid levels in Tg2576 mice with advanced amyloid accumulation.

**Figure 1:**
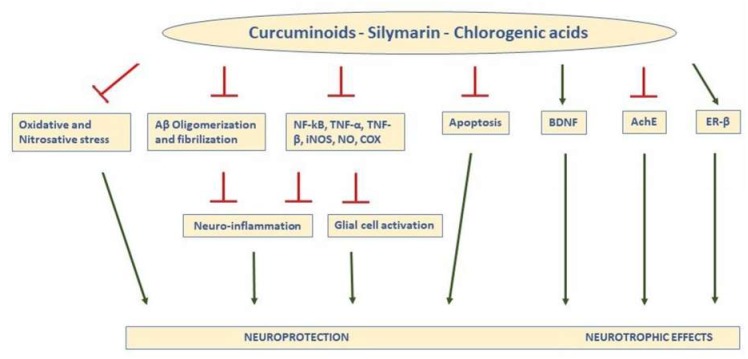
Schematic representation of the main actions responsible for the neuroprotective and neurotrophic effects of curcuminoids, silymarin, and Chlorogenic acids (CGA) in the brain. In general, the compounds inhibit oxidative and nitrosative stress, prevent formation of Aβ aggregates and fibrils, and reduce expression and activity of inflammatory agents, leading to less neuroinflammation and glial activation, decreasing apoptosis, increasing BDNF, inhibiting AChE, and binding ER-β, (T indicates inhibitory effects). TNF-a: tumor necrosis factor-a; NFkB: nuclear factor kappa light-chain enhancer of activated B cells; TNF-β, tumor necrosis factor-β; iNOS: inducible nitric oxide synthase; NO: nitric oxide; COX: cyclooxygenase; ER-β: estrogen receptor-β; BDNF: brain-derived neurotrophic factor; AChE: acetylcholinesterase.

A recent study analyzed the effects of a natural dietary supplement (NDS) containing *Curcuma longa* and other plant phenolic extracts, such as silymarin, guggul, chlorogenic acid, and inulin, on dysmetabolism and neurodegeneration in the brains of a diet-induced obesity mouse model. After 16 weeks of a hyper-lipidic diet with NDS, ROS concentration, lipid peroxidation, and expression of oxidative stress markers (p-ERK, H-Oxy, i-NOS, and HSP60) were significantly reduced in the brains of NDS-treated HFD mice in comparison with untreated obese mice, suggesting antioxidant effects of NDS. In addition, NDS was able to prevent neuronal apoptosis. In fact, a tunnel assay showed that in brain cortical sections of NDS-treated HFD mice, the number of neurons with fragmented DNA (index of an impairment of cell survival) was significantly reduced compared to HFD untreated animals [57]. The beneficial effects of NDS are probably due to the well-documented anti-oxidative actions of curcumin, although a synergistic action of all polyphenolic compounds contained in NDS has to also be considered. In fact, antioxidant and neuroprotective effects have been reported for silymarin and chlorogenic acid as well [72,73]. Figure 1 summarizes the main actions responsible for the neuroprotective effects of curcuminoids.

Despite the beneficial effects of curcumin in animal models, the available clinical studies are not enough to confirm the protective effect of curcumin for a patient’s treatment.

Epidemiologic studies have suggested that large amounts of curcumin intake are related to better cognitive function in healthy elderly individuals and a lower incidence of AD [74,75]. Furthermore, the expression of Aβ_42_, an Aβ form mainly involved in the early pathogenesis of the disease, is strongly reduced in the cerebro-spinal fluid of AD patients eating a diet rich in curcumin [76].

Nevertheless, clinical trials revealing any beneficial effects of curcuminoid administration for the treatment of AD are few and controversial [77,78,79]. The negative findings seem to be related to the low bioavailability of curcuminoids and the poor design of the clinical trials. More clinical studies with larger sample sizes and longer treatment durations should be designed to evaluate the effects of new formulations of curcumin with better bioavailability.

### 2.2. Silymarin

Silymarin is a mixture of flavonolignans, flavonoids, and other polyphenolic compounds extracted from milk thistle (*Silybum marianum*), an annual or a biennial plant belonging to the Asteraceae family and widely cultivated in the Mediterranean region. Silymarin is considered a scavenger of free radicals [80], and it is widely used in the treatment of liver discomfort. Moreover, silymarin is capable of protecting the central nervous system against injury and memory impairment [81,82]. Evidence for the neuroprotective action of silymarin has been reported both in animal models of neurodegenerative diseases [83,84] (Table 1) and in neuronal and non-neuronal cellular models [85,86,87]. The ability to slow the progression of neurodegeneration was also tested in CL4176 *Caenorhabditis elegans*, a model for AD. The results demonstrated that silymarin treatment significantly reduced the expression of amyloid β-protein (Aβ1-42) in muscle tissues of *C. elegans* via enhancing resistance to oxidative stress [72]. In rats, silymarin administration can improve ethanol-induced learning deficits [88] or prevent lipopolysaccharide and oxidative stress induced-neurotoxicity [89]. In APP transgenic mice, chronic silymarin treatment (half a year) improved AD-like phenotypes; in fact, it significantly reduced the cerebral plaque burden and brain microglial activation associated with an improvement of the behavioral abnormalities induced by AD pathology [86]. The molecular pathway of the neuroprotective potential of silymarin has mainly been ascribed to its capacity to inhibit oxidative stress in the brain [84,89], but different mechanisms, such as inhibition of the inflammatory response linked to neurodegeneration [90], neurotropic effects [91], inhibition of apoptosis [92] and regulation of neurotransmitters [93], have also been involved (Figure 1). More specifically, silymarin has been reported to attenuate neuronal damage in the hippocampus of Aβ1-42-injected rats by upregulating the brain-derived neurotrophic factor (BDNF)/ tyrosine receptor kinase B (TrkB) pathway and attenuating neuronal apoptosis [91]. It can improve learning and memory in diabetic rats by increasing BDNF levels [94]. Moreover, several studies report that the underlying mechanisms of the silymarin-induced neuroprotective effects may be due to the fact of its estrogen-like activity [95] as well as its potential to bind and activate ER-β [83,96].

Multiple findings obtained via in vitro and in vivo studies suggest that silymarin’s neuroprotective activity may be due the presence of silibinin which is its major pharmacologically active compound. Silibinin is able to attenuate oxidative stress-induced brain dysfunctions because of its potent antioxidative activity mainly related to the scavenging of free radicals [97,98] and increase in cellular GSH content [99] and SOD levels [100]. In addition, silibinin can inhibit Aβ aggregation in vitro and protect SH-SY5Y neuroblastoma cells from injuries caused by Aβ1-42-induced oxidative stress through a decrease in H_2_O_2_ production [85]. Silibinin can inhibit the activation of aging-related proteins and excessive ROS production in D-galactose-induced senescent mice [101], and it can protect against Aβ-induced neurotoxicity [102]. In particular, silibinin treatment attenuates memory impairment in intracerebroventricularly Aβ25-35-injected mice by reducing nitrotyrosine, iNOS, and TNF-α levels, and by increasing GSH levels in the hippocampus [84,102]. In the brain of APP/PS1 Tg mice, silibinin can reduce the amount of soluble and insoluble aβ40 and aβ42 and decrease the deposition of amyloid plaque and promote neurogenesis. These effects are associated with the inhibition of acetylcholinesterase activity and improvement in learning and memory deficits [93]. A further study showed that silibinin treatment significantly reduced MDA, ROS, GSH, and nitrite levels, strictly associated with STZ-induced memory impairment, and strongly counteracted the increase of acetylcholinesterase (AChE) and the decrease of α-7nicotinic acetylcholine receptor mRNA expression in IC-STZ-mouse brains, suggesting that the beneficial effect may be due to the improvement in brain energy metabolism and cholinergic function [103]. Recently, silibinin and silymarin have been shown to alleviate memory impairment of transgenic APP/PS1 mice through regulation of the gut microbiota. In fact, silibinin and silymarin administration modified the relative abundance of several bacterial species associated with AD [104]. However, it is necessary to underline that there is a lack of translational research and clinical evidence for these promising flavonoids which could be useful for treating neurodegenerative diseases [105].

### 2.3. Chlorogenic Acids

Another polyphenolic substance with excellent antioxidant activity is chlorogenic acid (CA), the major phenolic component in coffee. Chlorogenic acid belongs to the family of chlorogenic acids (CGAs), phenolic acids derived from the esterification of cinnamic acids including caffeic, ferulic, and *p*-coumaric acids. Besides coffee, CGAs are widely present in beverages prepared from herbs, fruits, and vegetables. The health benefits of consuming coffee, tea, and vegetable juice may be linked, at least in part, to their content of CGAs. Chlorogenic acids exhibit antibacterial, antioxidant, and anti-inflammatory activities [106].

Different in vitro and in vivo studies have highlighted the ability of CA or its derivatives to counteract neurodegenerative events (Table 1).

In vitro, CA is able to protect different types of neuronal cells from apoptosis and induced- oxidative neurotoxicity by blocking intracellular ROS accumulation, GSH depletion, MAPK, BACE-1, and α-secretase activation [107,108]. Chlorogenic acid can also counteract neuronal apoptosis in rat brain by inhibiting AChE and butyrilcholinesterase (BChE) activity, responsible for cholinergic deficits associated with memory loss of AD [109,110], and reducing pro-oxidant (FeSO4, sodium nitroprusside and quinolinic acid)-induced MDA [111]. Han et colleagues [112] demonstrated that CGA also has neuroprotective effects on Aβ-treated neuroblastoma SH-SY5Y cells through the upregulation of phosphoglycerate kinase-1 (PGK1) expression and the activation of ATP production. Accordingly, CA and its derivatives, 4,5-di-*O*-caffeoylquinic acid (4,5-di-CQA) and 3,4,5-tri-*O*-caffeoylquinic acid (3,4,5-tri-CQA), are able to counteract the aggregation of Aβ42 and its neurotoxicity on human neuroblastoma SH-SY5Y cells. The results of this study showed that 4,5-di-CQA and 3,4,5-tri-CQA inhibited the aggregation of Aβ42 in a dose-dependent manner by suppressing the radical-mediated aggregation of Aβ42 [113].

The protective effects of CGA on brain health and cognitive impairment have been evaluated using AD animal models. Chang et colleagues [114] showed the alleviative effect of CA on AD pathogenesis in HF diet-induced hyper-insulinemic rats. In particular, administration of CA for 30 weeks significantly ameliorated memory and learning impairments, cerebral insulin signaling, β-amyloid accumulation, and synaptic plasticity [114]. Chlorogenic acid also exerts anti-amnesic activity in mice with scopolamine-induced learning and memory deficits via inhibition of AChE and MDA in the hippocampus and frontal cortex [115]. A recent work investigated whether treatment with coffee polyphenols (CPPs) was able to prevent or reduce progressive impairments in memory and cognitive function in APP/PS2 mice, a model of AD. The results showed that CPP treatment prevented cognitive dysfunction by reducing amyloid Aβ plaques in the hippocampus due to the disaggregation of the fibrillar Aβ species into Aβ peptides [116].

To date, few studies have analyzed the effects of CGA on human cognitive impairment. Epidemiological studies found that coffee consumption habits may reduce the risk of mild cognitive impairment and AD [117,118]. The healthy benefits seem to be linked to CA antioxidant and anti-inflammatory properties (Figure 1). One study investigated the relationships between coffee intake and in vivo AD pathologies, including cerebral Aβ deposition, the neurodegeneration of AD signature regions, and cerebral white matter hyper intensities. The results showed that lifetime coffee intake of ≥2 cups/day was significantly associated with a lower Aβ positivity compared to coffee intake of <2 cups/day, suggesting that higher lifetime coffee intake may contribute to lowering the risk of AD or related cognitive decline by reducing pathological cerebral amyloid deposition [119]. Similar results were achieved by Eskelinen et al. [120]. They observed that coffee drinkers at midlife had lower risk of dementia and AD later in life compared with those drinking no or only little coffee. Kato et al. [121] reported that six-month intake of CGAs improved attentional, executive, and memory functions in the elderly with complaints of subjective memory loss. It is interesting to note that CGAs may improve some of the cognitive functions included in the CNS Vital Signs (Cognitrax) score, such as motor speed, psychomotor speed, and executive functions, in healthy individuals [122]. However, human studies have mainly investigated if coffee consumption improves cognitive performance, but its components have not been studied individually.

## 3. New Frontiers in Nutritional Prevention: Microalgal-Derived Extracts

Another class of natural compounds with strong neuroprotective potential and which is relatively unexplored yet is represented by extracts and biologically active compounds derived from microalgal biomass [123].

Microalgae are unicellular photosynthetic micro-organisms, living in saline or freshwater environments. They exist as eukaryotes, similar to green algae, or prokaryotes, similar to cyanobacteria [124]. The blue-green algae *Spirulina*, *Chlorella*, and *Aphanizomenon flos aquae* (AFA) are unicellular prokaryotic microorganisms belonging to the Cyanobacteria phylum. They are excellent sources of various biologically active compounds such as sterols, PUFAs, proteins, carotenoids, and polysaccharides [125]. Several experimental investigations have highlighted the anti-inflammatory [126], anticancer [127], and antioxidant [128,129,130,131] properties of microalgal-derived extracts and different studies have investigated the neuroprotective potentials of microalgae compounds against AD, mainly focusing on oxidative stress or β-amyloid aggregation. It has been reported that *Chlorella vulgaris* attenuate lead-induced oxidative damage in rats’ brains via an increase SOD, CAT, and GPX expressions as well as a reduction of MDA levels [132] and that *Chlorella* sp. and *Spirulina* sp. extracts exhibit antioxidant protection in rats’ brain homogenates [133]. *Spirulina maxima* also scavenged hydroxyl radicals and inhibited lipid peroxidation thanks to its phenolic extracts [134]. Biochanin A, an isoflavone identified in *Chlorella vulgaris*, exhibited neuroprotective activity against Aβ-induced neurotoxicity in neuroblastoma cell culture [135].

The *Aphanizomenon flos aquae* Ralfs ex Born. & Flah. var. *flos aquae* (AFA) species is a peculiar variety of blue-green microalgae that lives and proliferates in the Upper Klamath Lake (Oregon, USA). Due to the volcanic origin of the lake, Klamath AFA contain a peculiar pattern of macro- and micronutrients. In particular, AFA extracts are a rich source of vitamin B12, essential for myelin formation; trace minerals [136], such as molybdenum and tungsten, important antioxidant enzymes cofactors [137,138]; and pigments, such as carotene, beta-carotene, chlorophylls, and phycocyanins with well-known antioxidant and anti-inflammatory [139] properties.

A recent in vitro study analyzed the effects of the Klamath AFA extract (Klamin^®^) on oxidative stress and neurodegeneration in a neuronal LAN5 cell model. The results showed that Klamin^®^ was able to inhibit neuronal toxicity induced by the oxidant agent tert-butylhydroperoxide (TBH). This protective effect was mediated by scavenging activity, because the treatment with Klamin^®^ significantly decreased ROS generation in TBH-treated cells. Klamin^®^ was also able to prevent TBH-induced mitochondrial dysfunction, suggesting that its antioxidant capability can be extended to mitochondrial ROS. Furthermore, Klamin^®^ exerted a protective role against neuronal toxicity induced by Aβ-peptide. In particular, AFA extract interfered with Aβ aggregation by inhibiting its extension, producing aggregates of shorter dimensions, and destabilizing the preformed fibrils. Furthermore, the AFA extract exerted anti-inflammatory activity consequent to Aβ toxic stimulus, decreasing the production of proinflammatory cytokines such as IL-6 and IL-1β [140,141].

## 4. Conclusions

In conclusion, dietary interventions can be helpful tools for preventing or reversing the course of neurodegenerative diseases. Polyphenols are able to exert positive effects on brain health by enhancing neuronal function. The mechanisms of action of curcuminoids, silymarin, and chlorogenic acid can provide a defense against many pathophysiological features of neurodegenerative diseases such as oxidative stress, mitochondrial dysfunction, neuroinflammation, and protein aggregation. These natural products have proven to be promising for neurodegeneration and AD therapy in preclinical studies. Currently, clinical trials have shown inconsistent results, suggesting that further studies are necessary to uncover their therapeutic potential. Clinical studies have probably failed to succeed because treatment starts when the patients are already in an advanced state of the disease. A nutritional approach may circumvent this problem. The supplementation of bioactive natural compounds may start at a young age, allowing both prevention and a delay in disease progression. However, their bioavailability in the brain, including the ability to cross the blood–brain barrier, is yet to be established and remains one of the main obstacles for the development of therapies.

## Figures and Tables

**Table 1:** Summary of the main findings on the potential beneficial effects of curcuminoids, silymarin, and chlorogenic acids against neurodegeneration.

Outcome	Type of Study	Natural Compounds
Prevention of neurodegeneration [57].	Mice	*Curcuma longa*, Silymarin, Guggul, Chlorogenic Acid, and Inulin
Enhancement of memory [67].	Rats	Curcuminoids
Disruption of existing plaques and restoration of distorted neuritis [68].	Mice	Curcumin
Prevention of amyloid-beta deposition and attenuation inflammation in brain [69].	Mice	Curcumin
Amelioration of cognitive deficits and neurodegeneration [70].	Rats	Curcumin
Reduction oxidative damage and amyloid pathology [71].	Mice	Curcumin
Improvement in cognitive function and lower incidence of Alzheimer’s disease (AD) [74].	Epidemiological study	Curcumin
Reduction of Aβ42 expression in the cerebro-spinal fluid [75].	Epidemiological study	Curcumin
Improvement of the behavioral symptoms in AD [78].	Epidemiological study	Curcumin
Neuroprotective effects by reduction of oxidative stress [83,84,89].	Rats and mice	Silymarin
Attenuation of amyloid β plaque burden and improvement of behavioral abnormalities [86].	Mice	Silymarin
Dopaminergic neuron protection through inhibiting microglia activation, inflammation, and apoptosis [87,90].	Rats	Silymarin
Prevention of social learning deficits [88].	Rats	Silymarin
Neuroprotection by upregulation of neurotrophic factors and attenuation of autophagy, oxidative stress, and apoptosis [91,92].	Rats	Silibinin
Downregulation of acetylcholinesterase (AChE) activity and Aβ aggregation [93].	Mice	Silibinin
Improvement in learning and memory by increasing the brain-derived neurotrophic factor BDNF levels [94].	Rats	Silymarin
Neuroprotective effect due to the estrogen-like activity through selective activation of ERβ [95,96].	Rats	Silymarin
Increase of the glutathione content [99].	Rats	Silymarin
Prevention of memory impairment by reducing oxidative stress and Aβ aggregation [85,102]	Mice	Silibinin
Protection against senescence by inhibiting NF-kappaB activation and ROS production [101].	Mice	Silymarin
Improvement of memory impairment by increasing brain energy metabolism and cholinergic functions [103].	Mice	Silibinin
Regulative effects on relative abundance of several key bacterial species involved in AD development [104].	Mice	Silymarin and Silibinin
Protective effect against AD. Pathogenesis via modulating cerebral insulin signaling, β-Amyloid accumulation, and synaptic plasticity [114].	Rats	Caffeic Acid
Neuroprotective effects via anti-acetylcholinesterase and anti-oxidative activities [115].	Mice	Chlorogenic Acid
Prevention of cognitive dysfunction and suppression of amyloid β plaques [116].	Mice	Chlorogenic Acid
Reduction of mild cognitive impairment and AD risk [117,118].	Epidemiological study	Coffee
Decrement of amyloid pathology [119].	Epidemiological study	Coffee
Lower risk of dementia and AD later in life [120].	Epidemiological study	Coffee
Improvement of attentional, executive, and memory functions [121].	Human	Chlorogenic Acid
Improvement of cognitive functions including motor speed, psychomotor speed, and executive functions [122].	Human	Chlorogenic Acid
